# Hipk Is a Critical Mediator of Stress-Induced Intestinal Hyperplasia via the Hippo Pathway in *Drosophila*

**DOI:** 10.3390/biology15131029

**Published:** 2026-06-28

**Authors:** Xiaojie Wu, Hyung Chul Lee, Changsoo Kim

**Affiliations:** School of Biological Sciences and Technology, Chonnam National University, Gwangju 61186, Republic of Korea; wuxiaojie102030@gmail.com

**Keywords:** *hipk*, *yki*, *hippo*, regeneration, intestinal stem cells, proliferation, *Drosophila*

## Abstract

The *Drosophila* midgut serves as a powerful model system for studying the mechanisms underlying stem cell hyperproliferation induced by gut damage and Yorkie (Yki) activation. Homeodomain-interacting protein kinase (Hipk) has recently been shown to regulate intestinal stem cell (ISC) proliferation and differentiation during normal homeostasis. Here, we show that Hipk is cell-autonomously required for ISC hyperproliferation stimulated by both gut damage and Yki activation. Progenitor-specific *hipk* knockdown abolished the ISC hyperproliferation and gut hyperplasia induced by dextran sulfate sodium (DSS)-mediated injury and Yki overexpression. Mechanistically, Hipk promotes Yki protein stability and nuclear accumulation in progenitors. This regulation is specific to wild-type Yki, as the constitutively active Yki^S168A^ mutant, which escapes Warts-mediated inhibition, is insensitive to Hipk depletion. These findings identify Hipk as a critical regulator of Yki activation during ISC hyperproliferation.

## Simple Summary

Intestinal stem cell (ISC) proliferation is accelerated upon severe gut damage and Yorkie (Yki) activation. Homeodomain-interacting protein kinase (Hipk) has recently been shown to regulate ISC proliferation and differentiation during normal homeostasis. In this study, we demonstrate that Hipk is required for ISC hyperproliferation induced by DSS treatment and Yki activation, acting through the regulation of Yki protein activity in intestinal progenitors.

## 1. Introduction

The *Drosophila* midgut provides an invaluable model system for investigating the plasticity of adult stem cells across homeostasis, regeneration, and tumorigenesis [1,2,3,4,5]. Intestinal stem cells (ISCs) are located basally in the gut epithelium, where they maintain extensive contact with the basement membrane [6,7]. This membrane is apposed to visceral muscle cells, which form a specialized niche that supports ISC proliferation, stemness, and differentiation [8,9,10,11]. Under homeostatic conditions, ISCs undergo asymmetric cell division to produce a self-renewed ISC and a transient, post-mitotic progenitor cell, either an enteroblast (EB) or an enteroendocrine (EE) precursor (pre-EE) [12,13,14]. EBs further differentiate into enterocytes (ECs), the absorptive cells that constitute the epithelial monolayer, while pre-EEs differentiate into hormone-producing EEs [13]. In this binary lineage decision, most ISCs adopt an EB fate to supply the major EC population, while a subset adopts a pre-EE fate [12,13,15]. ISC proliferation is tightly regulated to maintain gut homeostasis.

However, ISC proliferation is accelerated by injury, such as exposure to dextran sulfate sodium (DSS) and activation of Yki, an oncogene that produces tumors when aberrantly activated [4,6,16,17,18,19]. The Hippo (Mst1 and Mst2 in mammals) pathway is an evolutionarily conserved regulator of cell growth and organ size that functions by restricting Yorkie (Yki) (YAP and TAZ in mammals), a transcriptional co-activator [20,21,22,23]. The Hippo pathway is a critical regulator of intestinal homeostasis and regeneration [24,25,26,27,28,29,30]. The Ste20-like kinases Misshapen (Msn) and Hippo (Hpo) regulate the Warts-Yorkie (Wts-Yki) axis in progenitors and ECs, respectively [24,28,31,32]. Yki are enriched in progenitors, where Yki activity is tightly regulated under normal homeostatic conditions [27,28,29,32]. Gut damage by DSS relieves the suppression of Yki by the upstream kinase cascade [27,28]. Once dephosphorylated, active Yki translocates to the nucleus to drive the expression of genes that promote cell division, underlying the ISC hyperproliferation observed during regeneration. Correspondingly, DSS-stimulated ISC hyperproliferation is significantly reduced by progenitor-specific *yki* knockdown [27,28]. Conversely, progenitor-specific activation of Yki either through loss of upstream signaling or direct Yki overexpression is sufficient to phenocopy the ISC hyperproliferation induced by chemical injury [27,28].

Recent findings have characterized Homeodomain-interacting protein kinase (Hipk) as a critical regulator of ISCs during homeostatic progenitor proliferation [33]. Here, we show that Hipk is essential for DSS- and Yki-induced proliferation by regulating Yki activity.

## 2. Materials and Methods

### 2.1. Drosophila Stocks and Husbandry

Animals were maintained on a standard cornmeal diet (68 g dry yeast, 90 g sucrose, 43 g cornmeal, 9 g agar, 4.5 mL propionic acid, 1 g methyl-4-hydroxybenaoate per 1 L water) at 25 °C and 40% relative humidity under 12 h light/dark cycle conditions. Midguts of virgin females were used for experiments unless otherwise stated. *W^1118^* (Bloomington *Drosophila* Stock Center, Bloomington, IN, USA) was used as control for experiments. Temporal control of transgene expression using *tub-Gal80^ts^* (Bloomington *Drosophila* Stock Center) in adult flies was achieved by raising flies at 20 °C throughout development until three days after eclosion followed by shifting to 29 °C to induce *Gal4*-mediated transgene expression. The duration of *UAS* transgene (Bloomington *Drosophila* Stock Center) induction is stated in each figure. Flies were transferred to fresh medium every second day. The following lines were generous gifts from the colleagues in the fly community: *esg^ts^* refers to *esg-Gal4*, *UAS-GFP/Cyo*; *tub-Gal80^ts^* (a gift from Perrimon, Harvard University, Cambridge, MA, USA). *esg^TS^* refers to *esg-Gal4*; *tub-Gal80^ts^* (this study, Kwangju-si, Korea). *UAS-yki^S168A^:GFP*, *UAS-yki:GFP* (a gift from Irvine, New Brunswick, NJ, USA) and *UAS-HA-hipk* (a gift from Verheyen, Burnaby, BC, Canada). *UAS-Hipk RNAi (III)* and *UAS-Hipk RNAi (II)* were obtained from the Bloomington *Drosophila* Stock Center (BDSC). *UAS-Wts RNAi (III)* was acquired from Vienna *Drosophila* Resource Center (VDRC, Vienna, Austria).

### 2.2. Gut Regeneration Assay 

Female flies (1 day old) were raised at 20 °C for three days and then shifted to 29 °C for seven days. Then, the flies were fed for another two days at 29 °C with sucrose (5%), DSS (5%), or *Pe* (Korean Collection for Type Cultures (KCTC), Daejeon-Si, Korea), after which midgut dissection and immunohistochemical analysis were performed. In detail, flies were put in a vial containing a disc of filter paper (Fisher catalog number 11111, Waltham, MA, USA) wet with 5% sucrose solution or 5% dextran sulfate sodium (DSS, MB Biomedicals, Costa Mesa, CA, USA) dissolved in 5% sucrose, resting on fly food. For *Pe* infection, a bacterial pellet from overnight culture in LB broth at 30 °C was resuspended in 5% sucrose solution to OD_600_ = 200. One hundred μL of the resuspension was smeared onto a filter paper disc. To ensure efficient intake of DSS or *Pe*, flies were starved for six hours before transfer to vials containing DSS or *Pe*. Flies were transferred to fresh vials with newly prepared DSS or *Pe* every day.

### 2.3. Immunohistochemistry

Adult guts were dissected out into 1× phosphate-buffered saline (PBS) and fixed in 4% paraformaldehyde for 30 min at room temperature. Samples were then washed 2 × 15 min in 1× PBS with 0.3% Triton X-100 (PBST) and incubated in blocking solution (PBST with 5% normal goat serum) for 30 min at room temperature. Samples were incubated with primary antibodies diluted in blocking solution overnight at 4 °C, washed 2 × 15 min at room temperature in PBST, incubated with secondary antibodies diluted in blocking solution at room temperature for 2 h, and washed again 2 × 15 min with PBST. Finally, guts were stained with DAPI in blocking solution for 30 min at room temperature and mounted in Fluoromount-G^®^ (SouthernBiotech, Birmingham, AL, USA) on a glass slide. Primary antibodies were used with the following dilutions: rabbit anti-phospho-Histone H3 Ser10 (pH3) (06-570, 1:1000 Merck Millipore, Burlington, MA, USA), mouse anti-β-Galactosidase (Z3781, 1:400 Promega, Madison, WI, USA), mouse anti-Delta (C594.9B, 1:50 DSHB, Iowa City, IA, USA), and rabbit anti-eGFP (CAB4211, 1:400 Invitrogen, Waltham, MA, USA). Secondary antibodies were used with the following dilutions: Alexa Fluor 488-conjugated goat anti-rabbit (A11008, 1:800 Invitrogen), Alexa Fluor 488-conjugated goat anti-mouse (A11001, 1:800 Invitrogen), Alexa Fluor 555-conjugated donkey anti-mouse (A31570, 1:800 Invitrogen) and Alexa Fluor 555-conjugated goat anti-rabbit (A21429, 1:800 Invitrogen). Images were taken from the posterior R4 region of the midgut with Leica Application Suite X software (version 3.7.4; Leica confocal microscope system, Wetzlar, Germany). Images were processed using ImageJ/Fiji (version 1.54k; National Institutes of Health, Bethesda, MD, USA). 

### 2.4. Statistical Analysis 

For quantitative analysis of cell populations, z-stacks were acquired from 20,000 μm^2^ square areas in the R4 region of each mounted midgut. Z-stacks were converted to maximum intensity projections, and cell numbers were manually counted using the Fiji Cell Counter plugin (version 1.0) integrated within ImageJ/Fiji (version 1.54k). For pH3-positive cell quantification, tile z-stacks covering the entire posterior midgut were acquired. The number of pH3-positive cells were determined by manual counting following confocal imaging. Statistical analysis were performed using GraphPad Prism 6 software. Statistical significance was denoted as follows: ns (not significant) > 0.05; *, *p* < 0.05; **, *p* < 0.01; ***, *p* < 0.001, and ****, *p* < 0.0001. All representative images were independently replicated at least three times with consistent results.

## 3. Results

### 3.1. Hipk Is Required to Mediate DSS-Induced Hyperplasia

**Figure 1:**
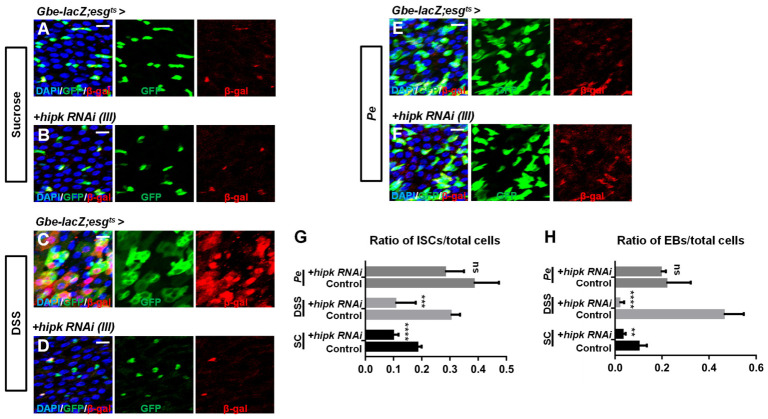
Hipk is required for DSS-induced progenitor cell expansion. (**A**–**F**) Representative confocal images of posterior midguts from control (*Gbe-lacZ*; *esg^ts^*) (**A**,**C**,**E**) and *hipk*-knockdown (*Gbe-lacZ*; *esg^ts^*, *UAS*-*hipk*-*RNAi*) (**B**,**D**,**F**) flies treated with sucrose (**A**,**B**), DSS (**C**,**D**), or *Pseudomonas entomophila* (*Pe*) (**E**,**F**) for two days at 29 °C. *esg^ts^* refers to *esg*-*Gal4*, *UAS-GFP*, *tub-Gal80^ts^*; *hipk RNAi* denotes *UAS*-*hipk RNAi*. GFP labels progenitor cells (*esg*-expressing cells); β-Gal labels EBs (*Gbe-lacZ*, a reporter for EBs); DAPI labels nuclei. Scale bars: 10 µm. Note that the progenitor population in DSS-treated midguts is significantly reduced upon *hipk* knockdown (compare **C** and **D**). (**G**,**H**) Quantification of the ratios of ISCs to total cells (**G**) and EBs to total cells (**H**) in the midguts of the indicated genotypes (mean ± SEM, *n* = 5). *p*-values were determined by two-tailed unpaired *t*-test: ns (not significant), *p* > 0.05; **, *p* < 0.01; ***, *p* < 0.001; ****, *p* < 0.0001.

To evaluate the role of Hipk in DSS- and Yki-induced gut hyperplasia, we used the temperature-sensitive, progenitor-specific *esg^ts^* driver (*esg-Gal4*, *UAS-GFP*; *tub-Gal80^ts^*) in combination with two distinct *UAS-hipk RNAi* lines (*UAS-hipk RNAi (II)* and *UAS-hipk RNAi (III)*) and *UAS-hipk* line; these lines were effectively used for *hipk* knockdown and *hipk* overexpression, respectivley, in progenitors [33]. The *esg^ts^* system enables temporal control of transgene expression in progenitor cells through a temperature shift from 20 °C to 29 °C. Progenitors, which consist of intestinal stem cells (ISCs) and enteroblasts (EBs), are dispersed among epithelial enterocytes (ECs) and characterized by *esg* expression (Figure S1A). All confocal images presented in this manuscript were obtained from the R4 region of the posterior midgut (Figure S1B). We used the *Su(H)Gbe-lacZ*; *esg^ts^* (*esg-Gal4*, *UAS-GFP*; *tub-Gal80^ts^*) reporter line to distinguish between ISCs (*GFP*^+^, *lacZ*^−^) and EBs (*GFP*^+^, *lacZ*^+^). In control midguts (*Su(H)Gbe-lacZ*; *esg^ts^*), the progenitor population (*GFP*^+^ cells) increased following two days of DSS treatment (Figure 1A,C), accompanied by the formation of large progenitor clusters (averaging ~5 cells per cluster; Figure 1C), which is indicative of accelerated ISC proliferation. Notably, the EB population showed a dramatic increase (from 10% to 50%), whereas the ISC population increased more modestly (from 20% to 30%) (Figure 1G,H). Progenitor-specific *hipk* knockdown (*Su(H)Gbe-lacZ*; *esg^ts^*/*UAS-hipk RNAi*) significantly suppressed this DSS-induced expansion and abolished the formation of progenitor clusters (Figure 1C,D). In particular, *hipk* depletion resulted in a marked decrease in the EB population (from 50% to 2%) and a moderate decrease in the ISC population (from 30% to 10%) (Figure 1G,H). In contrast, hyperplasia induced by *Pseudomonas entomophila* (*Pe*) infection was not significantly attenuated by *hipk* knockdown (Figure 1E–H), suggesting that Hipk-mediated regulation is specific to DSS-induced injury rather than *Pe*-stimulated damage.

### 3.2. Hipk Is Essential for Yki-Driven Hyperplasia

**Figure 2:**
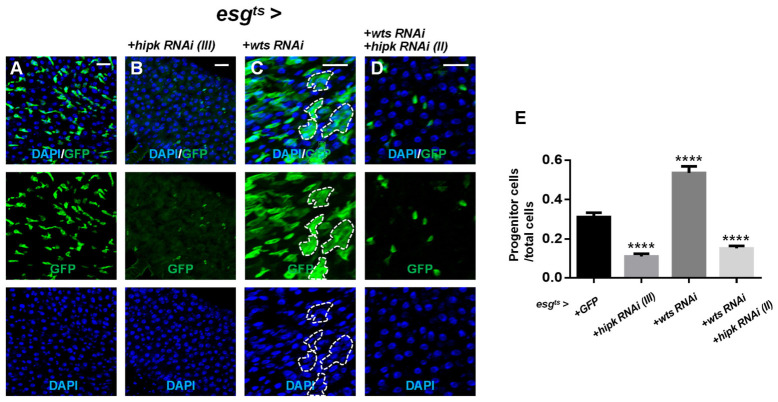
Hipk is required for *wts*-knockdown-stimulated progenitor expansion. (**A**–**D**) Representative confocal images of posterior midguts from control (*esg^ts^* > *UAS*-*GFP*) (**A**), *hipk*-knockdown (*esg^ts^* > *UAS*-*hipk RNAi*) (**B**), *wts* knockdown (*esg^ts^* > *UAS*-*wts RNAi*) (**C**), and *wts* and *hipk* co-knockdown (*esg^ts^* > *UAS*-*wts RNAi* + *UAS*-*hipk RNAi*) (**D**). Flies were treated for 7 days at 29 °C. The *esg^ts^* driver denotes *esg*-*Gal4*, *UAS-GFP*, *tub*-*Gal80^ts^*. Progenitor cells (*esg*-expressing cells) are marked by GFP expression; nuclei are stained with DAPI. Note that the progenitor expansion induced by *wts* knockdown is suppressed by the co-depletion of *hipk* (compare **C** and **D**). *wts* knockdown induces clusters of cells comprising ~5 cells, which are encircled (**C**). Scale bars: 20 µm. (**E**) Quantification of progenitor cell (ISC/EB) frequency in the midguts of the indicated genotypes (*n* = 9 per condition). Data are presented as mean ± SEM. *p*-values were determined by two-tailed unpaired *t*-test (****, *p* < 0.0001).

Yki activation in progenitor cells promotes hyperproliferation in ISCs, resulting in intestinal hyperplasia [27,28]. We next tested whether Hipk is required for Yki-induced ISC hyperproliferation. Consistent with previous studies, Yki activation via *wts* knockdown in progenitors (*esg^ts^* > *UAS*-*wts* *RNAi*) increased the progenitor population and induced tumor-like clusters (Figure 2A,C) and ISC proliferation (Figure S1C). Concurrent expression of *wts* *RNAi* and *hipk* *RNAi* in progenitors (*esg^ts^* > *UAS*-*wts* *RNAi* + *UAS*-*hipk* *RNAi*) reduced the progenitor population and eliminated these clusters (Figure 2C,D) and mitotic cells (Figure S1C), indicating that the hyperplasia and ISC hyperproliferation induced by *wts* knockdown requires Hipk.

**Figure 3:**
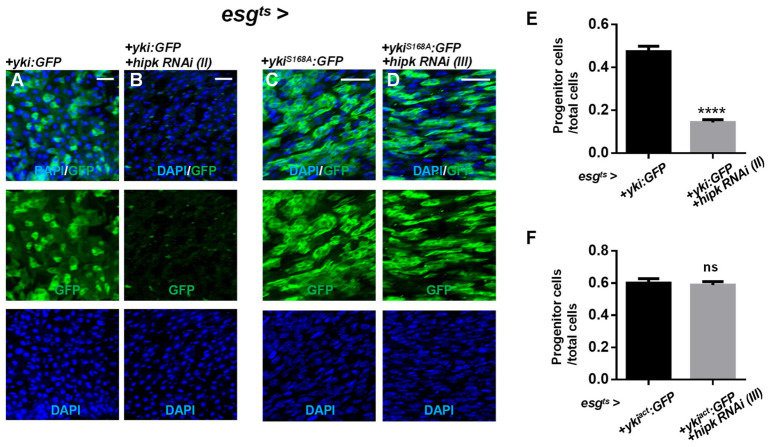
Hipk is required for Yki-stimulated progenitor expansion but is dispensable for Yki^S168A^-induced hyperplasia. (**A**–**D**) Representative confocal images of posterior midguts expressing in progenitors: *Yki* (*UAS-yki:GFP*) (**A**), *yki* and *hipk*-*RNAi* (*UAS-yki:GFP* + *hipk-RNAi*) (**B**), *yki^S168A^* (*UAS-yki^S168A^:GFP*) (**C**), and *yki^S168A^* and *hipk*-*RNAi* (*UAS-yki^S168A^:GFP* + *hipk*-*RNAi*) (**D**). *esg^ts^* refers to *esg*-*Gal4*, *tub*-*Gal80^ts^*, *UAS*-*GFP*. Flies were treated for 7 days at 29 °C. GFP marks progenitor cells (*esg*-expressing cells); DAPI marks nuclei. Note that the progenitor population induced by Yki:GFP is reduced by concomitant *hipk* knockdown (**B**), whereas the population induced by the constitutively active form, *yki^S168A^:GFP*, remains unaffected by *hipk* knockdown (**D**). Scale bar: 20 µm. (**E**,**F**) Quantification of progenitor cell (ISC/EB) frequency in the midguts of the indicated genotypes (*n* = 9 per condition). Data are presented as mean ± SEM. *p*-values were determined by two-tailed unpaired *t*-test (ns, *p* > 0.05; ****, *p* < 0.0001).

Similarly, hyperplasia and ISC hyperproliferation induced by progenitor-specific Yki overexpression (*esg^ts^* > *UAS-yki:GFP*) were suppressed by *hipk* knockdown (Figure 3A,B, Figure S1D). The Yki^S168A^ mutant is a constitutively active form refractory to Wts-mediated inhibition [34,35,36]. Strikingly, Yki^S168A^-mediated hyperplasia was not inhibited by concomitant *hipk* knockdown (*esg^ts^* > *UAS*-*yki^S168A^:GFP* + *UAS-hipk* *RNAi*) (Figure 3C,D), nor was mitotic activity reduced (Figure S1E).

### 3.3. Hipk Regulates Both Yki Protein Stability and Nuclear Localization

**Figure 4:**
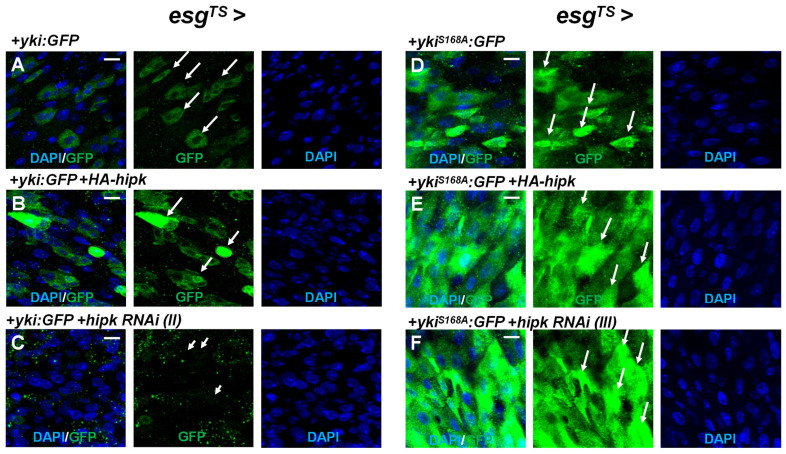
Hipk regulates the stability of wild-type Yki protein but is dispensable for the constitutively active Yki^S168A^ protein. (**A**–**F**) Representative confocal images of posterior midguts expressing in progenitors: *yki* (*UAS-yki:GFP*) (**A**), *yki* and *hipk* (*UAS-yki:GFP* + *UAS-hipk*) (**B**), *yki* and *hipk RNAi* (*UAS-yki:GFP* + *hipk RNAi*) (**C**), *yki^S168A^* (*UAS-yki^S168A^*:*GFP*) (**D**), *yki^S168A^* and *hipk* (*UAS*-*yki^S168A^:GFP* + *UAS-hipk*) (**E**), and *yki^S168A^* and *hipk RNAi* (*UAS*-y*ki^S168A^:GFP* + *hipk RNAi*) (**F**). *UAS*-transgenes were expressed for 7 days at 29 °C. The *esg^TS^* driver (*esg-Gal4*, *tub-Gal80^ts^*) was used without the *UAS-GFP* reporter to allow visualization of Yki::GFP and Yki^S168A^::GFP protein levels and localization. In control cells, Yki::GFP is predominantly cytoplasmic ((**A**), arrows). Conversely, Yki::GFP is elevated and localized to the nucleus upon *hipk* overexpression ((**B**), arrows), whereas it is undetectable in *hipk*-knockdown progenitors ((**C**), arrows). In contrast, Yki^S168A^::GFP is elevated and primarily nuclear-localized in control cells ((**D**), arrows). Neither *hipk* overexpression ((**E**), arrows) nor *hipk* knockdown ((**F**), arrows) alters the protein level or nuclear localization of Yki^S168A^::GFP. DAPI marks nuclei. Scale bars: 10 µm.

The differential effects of Hipk on Yki and Yki^S168A^ suggest that Hipk regulates the stability or localization of wild-type Yki, but not that of the constitutively active mutant. To test this hypothesis, we utilized the *esg^TS^* driver (*esg-Gal4*, *tub-GAL80^ts^*; lacking *UAS-GFP*) to express *UAS-yki:GFP* or *UAS-yki^S168A^:GFP*, where the GFP signal serves as a proxy for Yki::GFP or Yki^S168A^::GFP protein levels in progenitors. In control progenitors, Yki::GFP signals were predominantly detectable in the cytoplasm (Figure 4A). Hipk overexpression elevated Yki::GFP levels and promoted its nuclear localization (Figure 4A,B), whereas *hipk* knockdown rendered the Yki::GFP signal virtually undetectable (Figure 4A,C).

In contrast, the signals from Yki^S168A^::GFP were localized primarily to the nucleus and remained robust, showing no response to *hipk* manipulation (Figure 4D–F). These results demonstrate that Hipk is essential for the stability and nuclear accumulation of wild-type Yki, but is dispensable for Yki^S168A^.

## 4. Discussion

Hipk has recently been identified as a regulator of ISC proliferation and progenitor populations during normal intestinal homeostasis [33]. Here, we show that Hipk is required for DSS- and Yki-activated ISC hyperproliferation and progenitor expansion.

Dextran sulfate sodium (DSS)-activated ISC proliferation and progenitor expansion require Yki activity in progenitors [27,28]. Consistently, progenitor-specific Yki activation phenocopies the hyperplasia observed after DSS injury [27,28]. Here, we demonstrate that Hipk is required for both DSS- and Yki-induced ISC hyperproliferation and gut hyperplasia. Strikingly, Hipk is dispensable for Yki^S168A^-induced ISC hyperproliferation and gut hyperplasia. When Yki::GFP is overexpressed using the *esg^TS^* driver (*esg^TS^* > *UAS-yki::GFP*), the protein is weakly detectable in progenitors and predominantly sequestered in the cytoplasm, confirming active Hippo signaling under homeostatic conditions, consistent with previous reports [27,28]. Remarkably, Yki::GFP levels are further reduced, becoming essentially undetectable, upon *hipk* knockdown, whereas *hipk* overexpression increases Yki::GFP protein levels and promotes its nuclear localization. These findings indicate that Yki protein stability and nuclear accumulation are Hipk-dependent in progenitors. In contrast, when the constitutively active Yki^S168A^::GFP is overexpressed (*esg^TS^* > *UAS-yki^S168A^:GFP*), protein levels remain high and predominantly nuclear, indicating resistance to Hippo signaling. Notably, *hipk* manipulation does not affect the abundance or localization of Yki^S168A^::GFP, demonstrating that Hipk is dispensable for the activation of this mutant.

Yki^S168A^ is a mutant allele in which serine 168 is replaced by alanine, a non-phosphorylatable amino acid [36]. Ser168 is the critical Wts phosphorylation site; its phosphorylation by Wts triggers Yki cytoplasmic sequestration and degradation [34,35,36,37,38]. Recent evidence suggests that Hipk phosphorylates Yki at Ser169 and Ser172, residues proximal to the Ser168 target site [39]. Our data suggest that Hipk-dependent phosphorylation at Ser169 and Ser172 may sterically or chemically inhibit Wts-mediated phosphorylation at Ser168, thereby relieving Wts-dependent suppression, stabilizing Yki, and promoting nuclear translocation. This model accounts for why Hipk is dispensable for Yki^S168A^, which is inherently refractory to Ser168 phosphorylation (Figure S2). Previous studies have established that phosphorylation at Ser169 and Ser172 modulates Yki activity [39]. While phosphomimetic mutations (S169D/S172D) reduce Ser168 phosphorylation in *S2* cells and enhance Yki activity in vivo, alanine substitutions (S169A/S172A), mimicking a lack of Hipk phosphorylation, increase Ser168 phosphorylation in *S2* cells and suppress Yki activity in vivo [36].

The conservation of serine residues at positions +1 and +4 relative to Ser168 (e.g., Ser169 and Ser172 in *Drosophila* Yki) across both Yki and its mammalian homolog, YAP, suggests that mammalian Hipk may regulate YAP through a similar mechanism. Indeed, mammalian Hipk2 increases YAP abundance in cultured cells, suggesting an evolutionarily conserved regulatory axis [40]. Given the role of YAP in mammalian intestinal biology [41,42,43], our findings suggest that mammalian Hipk2 may similarly regulate intestinal hyperplasia, a process frequently associated with tumorigenesis.

Our data further show that *hipk* knockdown reduces DSS-stimulated progenitor expansion but fails to suppress expansion following infection by the bacterium *Pseudomonas entomophila* (*Pe*). This differential requirement for Hipk parallels that of Yki [28]. DSS activates Yki, as evidenced by nuclear localization, whereas *Pe* infection activates MAPK signaling in progenitors, indicated by elevated dpERK [27,44]. Specifically, DSS-induced ISC hyperproliferation is abolished by *yki* knockdown, whereas *Pe*-induced hyperproliferation is abolished by *egfr* knockdown [27,28,44]. Consistent with this, *hipk* expression does not require EGFR signaling, nor does forced EGFR activation affect *hipk* expression [33]. This explains why Hipk is required for DSS-stimulated, but not *Pe*-stimulated, ISC proliferation. However, further validation is required to determine whether MAPK signaling in response to *Pe* infection is indeed independent of Hipk in progenitors.

It is worth noting the emerging role of Hipk in the Hippo pathway during intestinal homeostasis and regeneration. We recently reported that *hipk* mutant clones typically consist of only one or two cells, whereas wild-type clones consistently generate more than ten cells within the same period, indicating a deficit in ISC proliferation [33]. In this study, we show that *hipk* depletion renders Yki protein undetectable, in stark contrast to wild-type progenitors where Yki is expressed, albeit predominantly sequestered in the cytoplasm. These findings suggest that the reduced proliferative capacity of ISCs following *hipk* knockdown under homeostatic conditions observed previously [33] is, in part, attributable to the destabilization of Yki, a well-established driver of cell proliferation. Furthermore, our observation that DSS-induced and Yki-driven ISC hyperproliferation is abolished by *hipk* knockdown can be explained by the depletion of Yki in the absence of Hipk. In a previous report [33], we showed that the impaired progenitor population during normal homeostasis observed upon *hipk* knockdown is not due to increased apoptotic cell death, but rather a loss of proliferative capacity; this is consistent with our current finding that Yki is depleted in *hipk*-deficient progenitors.

Although this study establishes a novel regulatory connection, a detailed epistatic analysis of the genetic interactions between *hipk* and the Hippo pathway during basal and regenerative homeostasis remains to be conducted. Given that *hipk* expression is governed by nutritional status via the *InR-Akt-mTOR* pathway [33], and that progenitor-specific Yki expression modulates nutrient signaling components [45], our finding that Hipk regulates Yki stability suggests that these pathways may be linked via Hipk to coordinate ISC proliferation. While the data presented here establish this connection, further rigorous genetic and molecular analyses are required to fully delineate the underlying mechanism.

## 5. Conclusions

Recent research indicates that Hipk phosphorylates Yki at sites proximal to the critical Wts-target residue. Here, we demonstrate that Hipk is essential for maintaining Yki protein stability in *Drosophila* midgut progenitors. Hipk depletion leads to a loss of Yki protein and abolishes Yki-induced intestinal hyperplasia. As aberrant Yki activity is a known driver of tumorigenesis, these results suggest that targeting Hipk kinase activity represents a promising therapeutic approach for managing Yki-associated intestinal cancers.

## Data Availability

Data supporting the results of this study are available from the corresponding authors on reasonable request.

## Supplementary Materials

The following supporting information can be downloaded at: https://www.mdpi.com/article/10.3390/biology15131029/s1, Figure S1: (A) Superficial view of the Drosophila midgut. (B) The Drosophila midgut is subdivided into several regions (R1–R5). (C–E) Quantification of pH3+ cells (mitotic cells) in midguts of the indicated genotypes. Figure S2: A model illustrating Hipk regulation of Yki activity.

## References

[1] Yu Z., Zhu Y., Chen Y., Feng C., Zhang Z., Guo X., Chen H., Liu X., Yuan Y., Chen H. (2024). Nutrient-sensing alteration leads to age-associated distortion of intestinal stem cell differentiating direction. Nat. Commun..

[2] Guo Z., Lucchetta E., Rafel N., Ohlstein B. (2016). Maintenance of the adult *Drosophila* intestine: All roads lead to homeostasis. Curr. Opin. Genet. Dev..

[3] Jiang H., Tian A., Jiang J. (2016). Intestinal stem cell response to injury: Lessons from *Drosophila*. Cell. Mol. Life Sci..

[4] Jiang H., Edgar B.A. (2011). Intestinal stem cells in the adult *Drosophila* midgut. Exp. Cell Res..

[5] Zhou J., Boutros M. (2023). Intestinal stem cells and their niches in homeostasis and disease. Cells Dev..

[6] Micchelli C.A., Perrimon N. (2006). Evidence that stem cells reside in the adult *Drosophila* midgut epithelium. Nature.

[7] Ohlstein B., Spradling A. (2006). The adult *Drosophila* posterior midgut is maintained by pluripotent stem cells. Nature.

[8] O’Brien L.E., Soliman S.S., Li X., Bilder D. (2011). Altered modes of stem cell division drive adaptive intestinal growth. Cell.

[9] Lin G., Xi R. (2008). Intestinal stem cell, muscular niche and Wingless signaling. Fly.

[10] Joly A., Rousset R. (2020). Tissue Adaptation to Environmental Cues by Symmetric and Asymmetric Division Modes of Intestinal Stem Cells. Int. J. Mol. Sci..

[11] Biteau B., Jasper H. (2011). EGF signaling regulates the proliferation of intestinal stem cells in *Drosophila*. Development.

[12] Ohlstein B., Spradling A. (2007). Multipotent *Drosophila* intestinal stem cells specify daughter cell fates by differential notch signaling. Science.

[13] Zeng X., Hou S.X. (2015). Enteroendocrine cells are generated from stem cells through a distinct progenitor in the adult *Drosophila* posterior midgut. Development.

[14] Li M., Tian A., Jiang J. (2024). Numb provides a fail-safe mechanism for intestinal stem cell self-renewal in adult *Drosophila* midgut. bioRxiv.

[15] de Navascues J., Perdigoto C.N., Bian Y., Schneider M.H., Bardin A.J., Martinez-Arias A., Simons B.D. (2012). *Drosophila* midgut homeostasis involves neutral competition between symmetrically dividing intestinal stem cells. EMBO J..

[16] Amcheslavsky A., Jiang J., Ip Y.T. (2009). Tissue damage-induced intestinal stem cell division in *Drosophila*. Cell Stem Cell.

[17] Biteau B., Hochmuth C.E., Jasper H. (2008). JNK activity in somatic stem cells causes loss of tissue homeostasis in the aging *Drosophila* gut. Cell Stem Cell.

[18] Choi N.H., Kim J.G., Yang D.J., Kim Y.S., Yoo M.A. (2008). Age-related changes in *Drosophila* midgut are associated with PVF2, a PDGF/VEGF-like growth factor. Aging Cell.

[19] Biteau B., Karpac J., Supoyo S., Degennaro M., Lehmann R., Jasper H. (2010). Lifespan extension by preserving proliferative homeostasis in *Drosophila*. PLoS Genet..

[20] Huang J., Wu S., Barrera J., Matthews K., Pan D. (2005). The Hippo signaling pathway coordinately regulates cell proliferation and apoptosis by inactivating Yorkie, the *Drosophila* Homolog of YAP. Cell.

[21] Pan D. (2010). The hippo signaling pathway in development and cancer. Dev. Cell.

[22] Staley B.K., Irvine K.D. (2012). Hippo signaling in *Drosophila*: Recent advances and insights. Dev. Dyn..

[23] Misra J.R., Irvine K.D. (2018). The Hippo Signaling Network and Its Biological Functions. Annu. Rev. Genet..

[24] Ma X., Guo X., Richardson H.E., Xu T., Xue L. (2018). POSH regulates Hippo signaling through ubiquitin-mediated expanded degradation. Proc. Natl. Acad. Sci. USA.

[25] Jiang D., Li P., Lu Y., Tao J., Hao X., Wang X., Wu W., Xu J., Zhang H., Li X. (2025). A feedback loop between Paxillin and Yorkie sustains *Drosophila* intestinal homeostasis and regeneration. Nat. Commun..

[26] Chen H.J., Li Q., Nirala N.K., Ip Y.T. (2020). The Snakeskin-Mesh Complex of Smooth Septate Junction Restricts Yorkie to Regulate Intestinal Homeostasis in *Drosophila*. Stem Cell Rep..

[27] Karpowicz P., Perez J., Perrimon N. (2010). The Hippo tumor suppressor pathway regulates intestinal stem cell regeneration. Development.

[28] Ren F., Wang B., Yue T., Yun E.Y., Ip Y.T., Jiang J. (2010). Hippo signaling regulates *Drosophila* intestine stem cell proliferation through multiple pathways. Proc. Natl. Acad. Sci..

[29] Shaw R.L., Kohlmaier A., Polesello C., Veelken C., Edgar B.A., Tapon N. (2010). The Hippo pathway regulates intestinal stem cell proliferation during *Drosophila* adult midgut regeneration. Development.

[30] Staley B.K., Irvine K.D. (2010). Warts and Yorkie mediate intestinal regeneration by influencing stem cell proliferation. Curr. Biol..

[31] Li Q., Li S., Mana-Capelli S., Roth Flach R.J., Danai L.V., Amcheslavsky A., Nie Y., Kaneko S., Yao X., Chen X. (2014). The conserved misshapen-warts-Yorkie pathway acts in enteroblasts to regulate intestinal stem cells in *Drosophila*. Dev. Cell.

[32] Li Q., Nirala N.K., Nie Y., Chen H.J., Ostroff G., Mao J., Wang Q., Xu L., Ip Y.T. (2018). Ingestion of Food Particles Regulates the Mechanosensing Misshapen-Yorkie Pathway in *Drosophila* Intestinal Growth. Dev. Cell.

[33] Wu X., Kim H., Jang W., Kim C. (2026). Hipk transduces nutrient signals to control intestinal stem cell proliferation and fate in *Drosophila*. Sci. Rep..

[34] Dong J., Feldmann G., Huang J., Wu S., Zhang N., Comerford S.A., Gayyed M.F., Anders R.A., Maitra A., Pan D. (2007). Elucidation of a universal size-control mechanism in *Drosophila* and mammals. Cell.

[35] Oh H., Irvine K.D. (2008). In vivo regulation of Yorkie phosphorylation and localization. Development.

[36] Oh H., Irvine K.D. (2009). In vivo analysis of Yorkie phosphorylation sites. Oncogene.

[37] Zhang L., Ren F., Zhang Q., Chen Y., Wang B., Jiang J. (2008). The TEAD/TEF family of transcription factor Scalloped mediates Hippo signaling in organ size control. Dev. Cell.

[38] Ren F., Zhang L., Jiang J. (2010). Hippo signaling regulates Yorkie nuclear localization and activity through 14-3-3 dependent and independent mechanisms. Dev. Biol..

[39] Steinmetz E.L., Dewald D.N., Walldorf U. (2021). *Drosophila* Homeodomain-Interacting Protein Kinase (Hipk) Phosphorylates the Hippo/Warts Signalling Effector Yorkie. Int. J. Mol. Sci..

[40] Poon C.L., Zhang X., Lin J.I., Manning S.A., Harvey K.F. (2012). Homeodomain-interacting protein kinase regulates Hippo pathway-dependent tissue growth. Curr. Biol..

[41] Kaur M., Mungurere R.F., Mitinje N., Sethi G.K., Kaur A.S., Mishra A. (2026). Hippo-YAP/TAZ signaling in gastric cancer: Molecular pathogenesis and emerging therapeutic horizons. Med. Oncol..

[42] Camargo F.D., Gokhale S., Johnnidis J.B., Fu D., Bell G.W., Jaenisch R., Brummelkamp T.R. (2007). YAP1 increases organ size and expands undifferentiated progenitor cells. Curr. Biol..

[43] Cao H., Huang X., Jiang X., Deng J., Wang J., Wu C., Hu M., Zeng B., Hu Z., Pan H. (2026). The WNK-OXSR1 osmosensing pathway mediates intestinal regeneration via Hippo-YAP signaling. EMBO J..

[44] Jiang H., Grenley M.O., Bravo M.J., Blumhagen R.Z., Edgar B.A. (2011). EGFR/Ras/MAPK signaling mediates adult midgut epithelial homeostasis and regeneration in *Drosophila*. Cell Stem Cell.

[45] Kwon Y., Song W., Droujinine I.A., Hu Y., Asara J.M., Perrimon N. (2015). Systemic organ wasting induced by localized expression of the secreted insulin/IGF antagonist ImpL2. Dev. Cell.

