# Supplementary material for: Hipk Is a Critical Mediator of Stress-Induced Intestinal Hyperplasia via the Hippo Pathway in Drosophila
Source: Biology (Basel). 2026 Jun 28;15(13):1029. doi: 10.3390/biology15131029 (PMC13360050; doi:10.3390/biology15131029)
Supplement: Supplementary file 1 [file biology-15-01029-s001.zip › biology-4366814-supplementary.pdf]

Communication

Hipk is a Critical Mediator of Stress-Induced Intestinal Hyperplasia via the Hippo Pathway in *Drosophila*

Xiaojie Wu [1], Hyung Chul Lee [1,*] and Changsoo Kim [1,*]

The transgenic flies used in this manuscript were validated as referenced.

Transgenic flies	References
UAS-Hipk RNAi (II)	Wu et al., scientific reports (2026) verheyen papers
UAS-Hipk RNAi (III)	Wu et al., scientific reports (2026) verheyen papers
UAS-Hipk	Wu et al., scientific reports (2026), verheyen papers
UAS-wts RNAi	Ren et al., PNAS (2010), Ding., Cell Death & Disease (2021), Chen., Cell Press (2012), Shaw et al., Development (2010)
UAS-Yki:GFP	Fletcher et al., Development (2018), Oh., Development (2008), Chen., Cell Press (2012), Shaw et al., Development (2010)
UAS-Yki^{S168A}:GFP	Oh., Development (2008), Chen., Cell Press (2012)

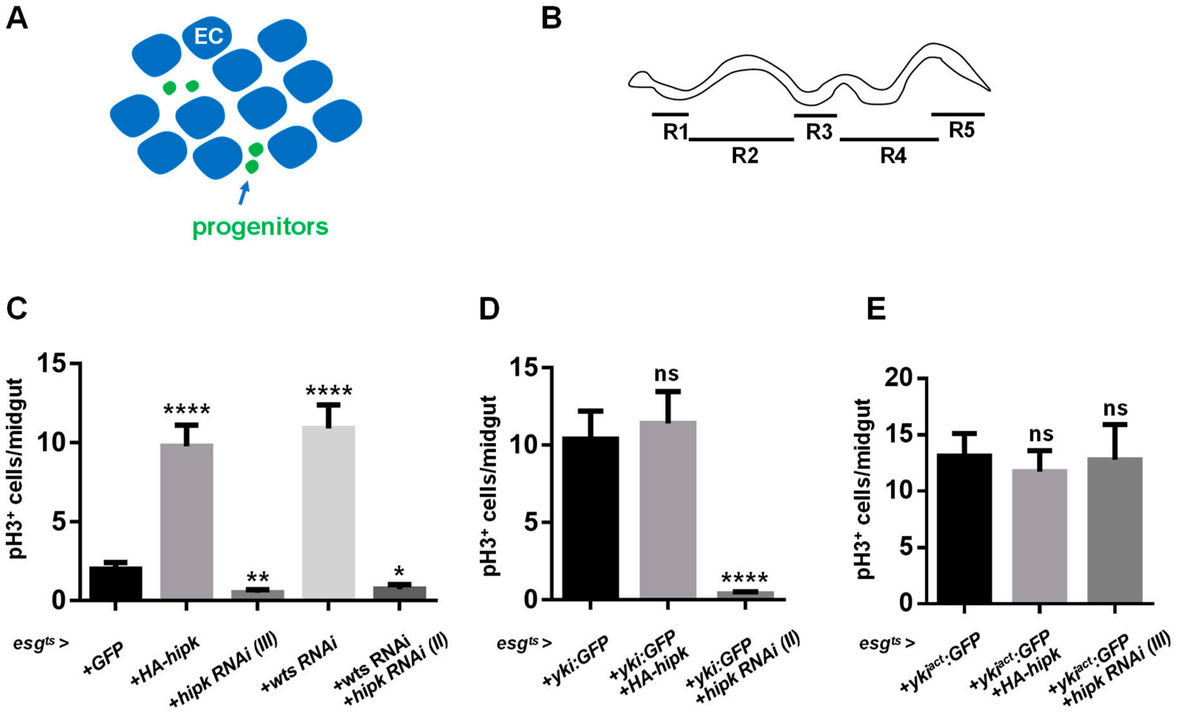

Figure S1. (A) Superficial view of the *Drosophila* midgut. The epithelium consists primarily of enterocytes

(ECs), which form a monolayer. Progenitors, comprising intestinal stem cells (ISCs) and enteroblasts (EBs), are scattered throughout the epithelium. EBs are transient, postmitotic cells that differentiate into ECs. (B) The *Drosophila* midgut is subdivided into several regions (R1–R5). Confocal images throughout this manuscript focus on the R4 region of the posterior midgut. (C-E) Quantification of pH3$^+$ cells (mitotic cells) in midguts of the indicated genotypes. UAS-transgenes were expressed using the *esg^{ts}* driver for 7 days at 29 °C. Guts were isolated and stained with anti-phospho-histone H3 antibody. Mitotic cells in whole midguts were counted for all genotypes. Error bars indicate mean ± SEM from n > 5 midguts for all genotypes. *P*-values were determined by two-tailed unpaired *t*-test: ns, $p > 0.05$; *, $p < 0.05$; **, $p < 0.01$; ****, $p < 0.0001$. The *esg^{ts}* driver denotes *esg-Gal4*, *UAS-GFP, tub-Gal80^{ts}*. (A) *+HA-hipk* denotes *UAS-hipk*, *+hipk-RNAi (III)* denotes *UAS-hipk RNAi (III)*, *+wts RNAi* denotes *UAS-wts RNAi*, and *+wts RNAi + hipk RNAi* denotes *UAS-wts RNAi + UAS-hipk RNAi* (II). (B) *+yki:GFP* denotes *UAS-yki:GFP*, *+yki:GFP + HA-hipk* denotes *UAS-yki:GFP + UAS-hipk*, *+yki:GFP + hipk RNAi (II)* denotes *UAS-yki:GFP + UAS-*hipk *RNAi (II)*. (C) *+yki^{act}:GFP* denotes *UAS-yki^{S168A}:GFP*, *+yki^{act}:GFP + HA-hipk* denotes *UAS-yki^{S168A}:GFP + UAS-hipk* and *+yki^{act}:GFP + hipk RNAi (III)* denotes *UAS-yki^{S168A}:GFP + UAS-hipk RNAi (III)*.

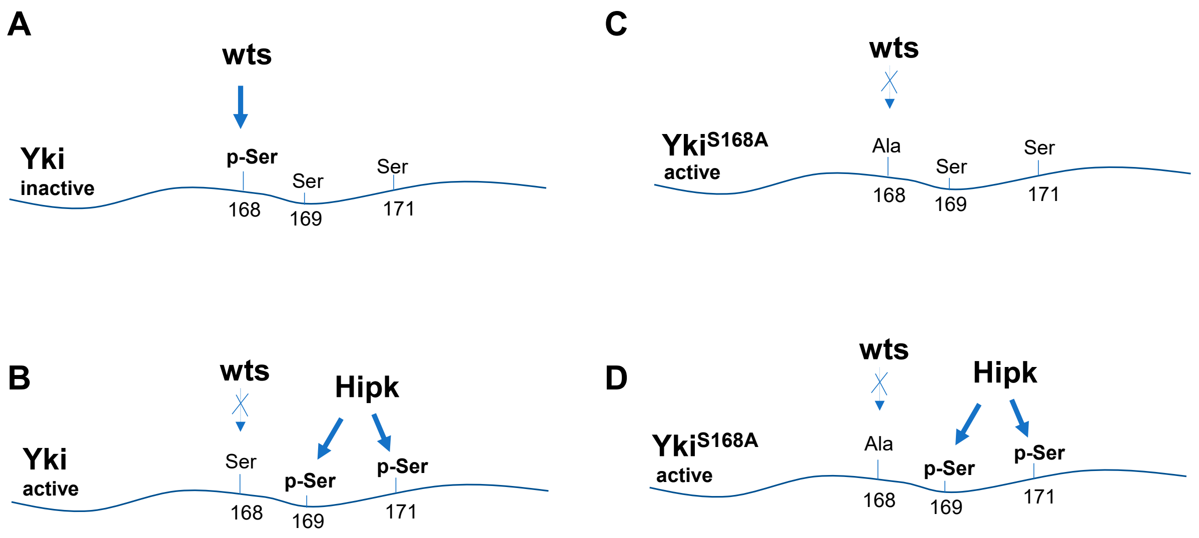

Figure S2. **A model illustrating Hipk regulation of Yki activity.** (A, B) Hipk phosphorylates Yki at Ser169 and Ser171 [39]. This study demonstrates that Hipk regulates wild-type Yki activity but is dispensable for the activity of the Yki^{S168A} mutant. We propose a model in which Hipk-mediated phosphorylation at Ser169 and Ser171 inhibits Wts-mediated phosphorylation of Yki at Ser168. Consequently, Hipk modulates the level of

Wts-dependent phosphorylation at Ser168, thereby tuning Yki activity. (C, D) Yki^{S168A}, which is refractory to Wts-mediated phosphorylation, remains constitutively active even in the presence of Wts. Furthermore, Yki^{S168A} activity is independent of Hipk-mediated phosphorylation at Ser169 and Ser171.
